# Alterations in resting-state functional connectivity after brain posterior lesions reflect the functionality of the visual system in hemianopic patients

**DOI:** 10.1007/s00429-022-02502-0

**Published:** 2022-05-19

**Authors:** Jessica Gallina, Marco Zanon, Ezequiel Mikulan, Mattia Pietrelli, Silvia Gambino, Agustín Ibáñez, Caterina Bertini

**Affiliations:** 1 Centre for Studies and Research in Cognitive Neuroscience, University of Bologna, Cesena, Italy; 2 Department of Psychology, University of Bologna, Bologna, Italy; 3 Neuroscience Area, International School for Advanced Studies (SISSA), Trieste, Italy; 4 Department of Biomedical and Clinical Sciences “L. Sacco”, University of Milan, Milan, Italy; 5 Department of Psychiatry, University of WI–Madison, Wisconsin, USA; 6 Latin American Brain Health (BrainLat), Universidad Adolfo Ibáñez, Santiago, Chile; 7 Cognitive Neuroscience Center (CNC), Universidad de San Andrés, Buenos Aires, Argentina; 8 National Scientific and Technical Research Council (CONICET), Buenos Aires, Argentina; 9 Global Brain Health Institute, University of California-San Francisco, San Francisco, CA USA; 10 Trinity College Dublin, Dublin, Ireland

**Keywords:** Functional connectivity, Alpha oscillations, Theta oscillations, Resting state, Hemianopia

## Abstract

Emerging evidence suggests a role of the posterior cortices in regulating alpha oscillatory activity and organizing low-level processing in non-alpha frequency bands. Therefore, posterior brain lesions, which damage the neural circuits of the visual system, might affect functional connectivity patterns of brain rhythms. To test this hypothesis, eyes-closed resting state EEG signal was acquired from patients with hemianopia with left and right posterior lesions, patients without hemianopia with more anterior lesions and healthy controls. Left-lesioned hemianopics showed reduced intrahemispheric connectivity in the range of upper alpha only in the lesioned hemisphere, whereas right-lesioned hemianopics exhibited reduced intrahemispheric alpha connectivity in both hemispheres. In terms of network topology, these impairments were characterized by reduced local functional segregation, with no associated change in global functional integration. This suggests a crucial role of posterior cortices in promoting functional connectivity in the range of alpha. Right-lesioned hemianopics revealed also additional impairments in the theta range, with increased connectivity in this frequency band, characterized by both increased local segregated activity and decreased global integration. This indicates that lesions to right posterior cortices lead to stronger impairments in alpha connectivity and induce additional alterations in local and global low-level processing, suggesting a specialization of the right hemisphere in generating alpha oscillations and in coordinating complex interplays with lower frequency bands. Importantly, hemianopic patient’s visual performance in the blind field was linked to alpha functional connectivity, corroborating the notion that alpha oscillatory patterns represent a biomarker of the integrity and the functioning of the underlying visual system.

## Introduction

Cognitive functioning is a distributed and dynamic process, requiring functional interactions among neural populations widely distributed in cortical and subcortical networks. Such interactions between local and remote brain regions have been effectively studied in the domain of EEG research by functional connectivity, measuring the statistical interdependencies between EEG rhythms in a condition of resting state, between different pairs of electrodes (Stam 2010; Aertsen et al. 1989). This electrophysiological marker of functional coupling is able to capture relationships among different brain regions, which are essential for brain functioning (Varela et al. 2001; Tononi and Edelman 1998). In line with this perspective, studies on the healthy brain have shown that spontaneous EEG fluctuations in the resting brain are typically highly organized and coherent (Greicius et al. 2003). More recent findings have also described the complexity of brain connectivity using graph theory (Strogatz 2001), a mathematical approach that quantifies topological properties of neural networks, defining a complex system of nodes (vertices) and edges (links), whose functional activity is characterized by a balance between local specialization and global integration (Ma et al. 2021; Stam, et al. 2007; Tononi, et al. 1994).

A variety of neurological (Parra et al. 2017; Dottori et al. 2017; Babiloni et al. 2008, 2016; Melloni et al. 2015; Rossini et al. 2007) and psychiatric (Barttfeld et al. 2014, 2013; Fingelkurts et al. 2007; Dawson 2004; Haig et al. 2000) conditions have demonstrated to be associated to alterations of the typical pattern of functional connectivity, suggesting that these indices might represent a reflection of neural integrity. In line, investigations in patients with brain lesions have shown a wide range of post-lesional changes in functional connectivity in different frequency bands. Changes in the low-frequency bands (delta/theta) were mainly reported in terms of an increase in the number (Castellanos et al. 2010) and the functionality (Dubovik et al. 2012; Castellanos et al. 2010) of the connections in patients with acquired brain lesions, compared to controls, whereas reports on post-lesional changes in higher frequency bands (e.g., beta) were rather scarce and mostly associated to task-related oscillatory activity (Guggisberg, et al. 2015). In contrast, various patterns of changes have been reported in the alpha range at rest, showing both a post-lesional reduction of brain connectivity, especially in the ipsilesional hemisphere (Dubovik et al. 2012; Westlake et al. 2012; Wu et al. 2011; Castellanos et al. 2010) and in interhemispheric interactions (Wu et al. 2011), but also increased alpha connectivity in the contralesional hemisphere (Westlake et al. 2012) or in the intact regions of the lesioned hemisphere (Wu et al. 2011; Guggisberg et al. 2008). Moreover, post-stroke reduced functional connectivity in the alpha band has also been related to more severe deficits in functional outcomes (Guggisberg et al. 2015; Dubovick et al. 2012). Similarly, graph theory analyses have also described rearrangements of network topology following stroke, disrupting the balance between local and global processing (Caliandro et al. 2017). More precisely, among others graph theoretical parameters, clustering coefficient (C), reflecting an index of functional segregation, and characteristic path length (L), measuring functional integration, have been shown to be often altered in patients with brain lesions (Vecchio et al. 2019a; 2019b; Caliandro et al. 2017), suggesting decreased functioning efficiency (Achard, et al. 2006).

Despite the increasing evidence of alterations of the electrophysiological functionality of brain networks after brain lesions, the variety of clinical and lesional profiles in the existing literature has concurred to provide only partial and unselective proofs of changes in post-lesional connectivity at rest so far. Indeed, recent findings, investigating oscillatory activity during resting state in hemianopic patients have suggested that posterior lesions, targeting the structures of the visual pathways and resulting in visual field defects (Grasso, et al. 2020a, b), might induce specific alterations in oscillatory patterns. More precisely, posterior brain lesions in hemianopic patients have been shown to selectively reduce the alpha peak and power during rest, while the same was not observed after lesions to anterior regions (Pietrelli et al. 2019), suggesting a role of posterior cortices in generating and distributing alpha oscillatory activity at rest. Moreover, evidence has shown that lesions to posterior cortices (and not lesions to anterior cortices) alter also the typical alpha power reduction observed in the transition from the eyes-closed resting state to the opening of the eyes (i.e., alpha reactivity; Gallina, et al. 2021). Interestingly, when alpha reactivity was more severely impaired after right posterior lesions, a concurrent disruption in reactivity in the theta range at the opening of the eyes was also observed (Gallina et al. 2021). This converging evidence suggests a role of posterior cortices in orchestrating these oscillatory patterns at rest, both coordinating widespread alpha oscillatory activity and organizing focal processing in the theta range and that lesions to these cortices and impairing the visual system might selectively disrupt this oscillatory activity. In line with this view, the relevance of posterior cortices in oscillatory activity, namely in the alpha range, has been widely reported. Occipito-parietal regions, indeed, typically show prominent oscillatory activity in the alpha range (7–13 Hz) during resting state (Rosanova et al. 2009) and have been reported to play a pivotal role in generating alpha oscillations (Thut et al. 2011; Bollimunta et al. 2008). In addition, activity in the alpha range has been reported to be linked to the excitability of the visual cortices (Romei et al. 2008a, b) and to be associated to aware (Pfurtscheller, et al. 1994) and unaware (Grasso, et al. 2020a, b) visual processing and visuo-spatial attention (Capilla, et al. 2014). In this perspective, oscillations in the alpha range have been suggested to reflect, even at rest, the activity of the underlying neural populations (Sadaghiani and Kleinschmidt 2016; Klimesch et al. 2007) and, thus, the functionality of the visual system. Moreover, alpha oscillations, propagating from posterior visual cortices to higher order cortical sites (Hindriks et al. 2015), might play a special role in coordinating widespread oscillatory activity and orchestrating focal processing in non-alpha frequency bands.

As a consequence, in light of the importance of posterior cortices in generating, distributing and coordinating oscillatory patterns (Hindriks et al. 2015; Thut et al. 2011; Bollimunta et al. 2008; Romei, et al. 2008a, b), it is of great interest to study the effects of lesions to these cortices on oscillations also at the network level. Indeed, as previously mentioned, evidence on hemianopic patients has shown that lesions to posterior cortices alter oscillatory activity at rest and that these alterations might represent a biomarker of the functionality of the visual system (Pietrelli et al. 2019; Gallina et al. 2021). However, it is still unknown whether lesions to posterior cortices might also induce specific post-lesional alterations of functional connectivity patterns in different frequency bands during resting state. Recent human fMRI studies on hemianopic patients showed a decrease in brain functional connectivity, particularly evident in the Visual Network (Pedersini et al. 2020), suggesting that lesions to posterior cortices might affect connectivity, but providing no insight on possible alterations in connectivity in specific frequency bands. Moreover, preliminary EEG investigations showed the presence of some altered patterns of connectivity in the alpha range (Guo et al. 2014; Wang et al. 2012) and suggested that impaired alpha connectivity at rest is predictive of the severity of the visual field deficits (Allaman et al. 2021) after lesions to the visual cortices, but the consequences of posterior lesions on the complex pattern of alpha and non-alpha (e.g. theta) oscillatory processing at rest still needs to be elucidated. Moreover, previous investigations have revealed a different impact of posterior left and right lesions on resting oscillations, with right-damaged hemianopics showing a more severe impairments in alpha peak and in the distributions of alpha power (Pietrelli et al. 2019) and a greater dysfunction in oscillatory reactivity at the opening of the eyes (Gallina et al. 2021). Therefore, the present study aims at investigating whether posterior lesions affect the complex pattern of functional connectivity in different frequency bands and whether lesions to the left or the right hemisphere can differentially alter connectivity patterns.

To explore these hypotheses, EEG activity during eyes-closed resting state was recorded in patients with left or right lesions to the posterior cortices, in control patients with left or right more anterior lesions and in a group of healthy controls. Intrahemispheric and interhemispheric connectivity indices were computed to measure post-lesional functional connectivity changes. In addition, clustering coefficient (C) and characteristic path length (L) were chosen as graph theory parameters to characterize local and global connectivity patterns, since they have been suggested to appropriately represent post-lesional alterations in network topology (Caliandro et al. 2017; Vecchio et al. 2019a, b). Connectivity indices and graph theoretical parameters were computed in theta (3–6 Hz), lower alpha (7–10 Hz), upper alpha (11–13 Hz) and beta (14–25 Hz) bands, to inspect a broad range of oscillations (Caliandro et. al. 2017). The lower and upper ranges of the alpha band were separately explored, to account for possible alterations in patients with posterior lesions, due to their previously documented slowdown in individual alpha frequency (Pietrelli et al. 2019). Finally, the relationship between visual performance in hemianopics and connectivity indices was also investigated, to explore whether specific connectivity alterations might be linked to visual impairments in hemianopics.

## Materials and methods

### Participants

Five groups of participants took part in the study: 14 patients (10 males, mean age = 53.08 years, SD = 15.32; mean education = 12.07 years, SD = 2.58; mean time since lesion onset = 12.64 months, SD = 11.59) with visual field defect due to lesions to the left posterior cortices, 13 patients with visual field defect due to lesions to the right posterior cortices (10 males, mean age = 58.9 years, SD = 16.47; mean education = 12 years, SD = 5.20; mean time since lesion onset = 12.47 months, SD = 14.18), a control group of nine patients without hemianopia with lesions to left fronto-temporal cortices, sparing the posterior cortices (5 males, mean age = 43.22 years, SD = 9.65; mean education = 14.67 years, SD = 2.69; mean time since lesion onset = 19.56 months, SD = 14.83), a control group of nine patients without hemianopia with lesions to right fronto-temporal cortices, sparing the posterior cortices (4 males, mean age = 51.67 years, SD = 9.97; mean education = 10.44 years, SD = 3.98; mean time since lesion onset = 23 months, SD = 25.15) and a control group of fourteen age-matched healthy participants (7 males, mean age 54.29 years, SD = 8.28; mean education = 13.28 years, SD = 3.12). No differences between the groups were found in terms of age (*F*_1,54_ = 2.12; *p* = 0.091), education (*F*_1,54_ = 1.761, *p* = 0.150) or time since lesion onset (*F*_1,41_ = 1.072; *p* = 0.371; for clinical details, please see Table 1).

**Table 1:** Summary of demographic and clinical data of all patients that took part to the study

Subject	Sex	Age	Education	Onset	Lesion site	Visual field defect	Aetiology
HEMI L-LES 1	M	69	11	5	Left-occipital	Right hemianopia	Ischaemic
HEMI L-LES 2	M	45	13	7	Left-temporal	Right hemianopia	Hemorragic
HEMI L-LES 3	F	57	13	28	Left fronto-temporo-insular	Right hemianopia	AVM
HEMI L-LES 4	M	50	13	7	Left temporo-occipito-parietal	Upper right quadrantopia	Ischaemic
HEMI L-LES 5	M	81	5	9	Left occipito-temporal	Right hemianopia	Abscess
HEMI L-LES 6	M	51	13	5	Left fronto-temporo-occipital	Right hemianopia	Ischaemic
HEMI L-LES 7	M	41	13	2	Left occipital	Lower right quadrantopia	Hemorragic
HEMI L-LES 8	M	45	13	42	Left fronto-parieto-temporal	Right hemianopia	AVM
HEMI L-LES 9	F	29	15	26	Left temporal	Upper right quadrantopia	Ischaemic
HEMI L-LES 10	M	58	8	6	Left temporo-occipital	Right hemianopia	Ischaemic
HEMI L-LES 11	F	32	16	4	Left parieto-occipital	Right hemianopia	Ischaemic
HEMI L-LES 12	M	69	13	8	Left temporo-occipital	Right hemianopia	Hemorragic
HEMI L-LES 13	M	73	12	17	Left temporo-occipital	Right hemianopia	Hemorragic
HEMI L-LES 14	F	59	11	11	Left-mesial-temporal	Right hemianopia	AVM
HEMI R-LES 1	M	56	18	3	Right occipital	Left hemianopia	Ischaemic
HEMI R-LES 2	F	38	18	13	Right parieto-occipital	Left hemianopia	Hemorragic
HEMI R-LES 3	F	37	13	4	Right occipito-temporo-parietal	Left hemianopia	Tumor
HEMI R-LES 4	M	58	8	18	Right temporo-occipital	Left hemianopia	Ischaemic
HEMI R-LES 5	M	81	8	7	Right occipital	Left hemianopia	Hemorragic
HEMI R-LES 6	M	51	8	4	Right occipital	Left hemianopia	Tumor
HEMI R-LES 7	M	60	18	29	Right temporo-occipital	Left hemianopia	Ischaemic
HEMI R-LES 8	F	73	5	8	Right temporo-occipital	Left hemianopia	Ischaemic
HEMI R-LES 9	M	77	8	6	Right fronto-parietal	Left hemianopia	Hemorragic
HEMI R-LES 10	M	30	16	54	Right-temporal	Left hemianopia	Hemorragic
HEMI R-LES 11	M	59	18	5	Right temporo-occipital	Left hemianopia	Ischaemic
HEMI R-LES 12	M	76	13	7	Right temporo-occipital	Left hemianopia	Abscess
HEMI R-LES 13	M	70	18	5	Right occipital	Left hemianopia	Ischaemic
CONT L-LES 1	F	48	13	38	Left fronto-insular	No hemianopia	Ischaemic
CONT L-LES 2	F	44	13	40	Left frontal	No hemianopia	Tumor
CONT L-LES 3	M	28	18	11	Left fronto-parietal	No hemianopia	Tumor
CONT L-LES 4	F	45	11	39	Left frontal	No hemianopia	Tumor
CONT L-LES 5	F	46	15	12	Left temporal pole	No hemianopia	Hemorragic
CONT L-LES 6	M	62	18	7	Left temporo-insular	No hemianopia	Abscess
CONT L-LES 7	M	34	13	7	Left frontal	No hemianopia	Tumor
CONT L-LES 8	M	45	13	15	Left-frontal	No hemianopia	Ischaemic
CONT L-LES 9	F	37	13	7	Left frontal	No hemianopia	Tumor
CONT R-LES 1	F	57	13	5	Right fronto-insular	No hemianopia	AVM
CONT R-LES 2	M	42	18	59	Right frontal	No hemianopia	Abscess
CONT R-LES 3	F	42	11	19	Right frontal	No hemianopia	Tumor
CONT R-LES 4	M	51	8	3	Right temporo-insular	No hemianopia	Tumor
CONT R-LES 5	F	51	10	5	Right temporal	No hemianopia	Tumor
CONT R-LES 6	F	50	5	71	Right temporo-fronto-polar	No hemianopia	Traumatic
CONT R-LES 7	M	75	8	26	Right temporo-insular	No hemianopia	Tumor
CONT R-LES 8	F	46	8	13	Right-frontal	No hemianopia	Abscess
CONT R-LES 9	M	51	13	6	Right-frontal	No hemianopia	Tumor

Details about age and education are reported in years; details about onset of brain lesion are reported in months

*M* Male, *F* Female, *AVM* Arteriovenus malformation

**Fig. 1:**
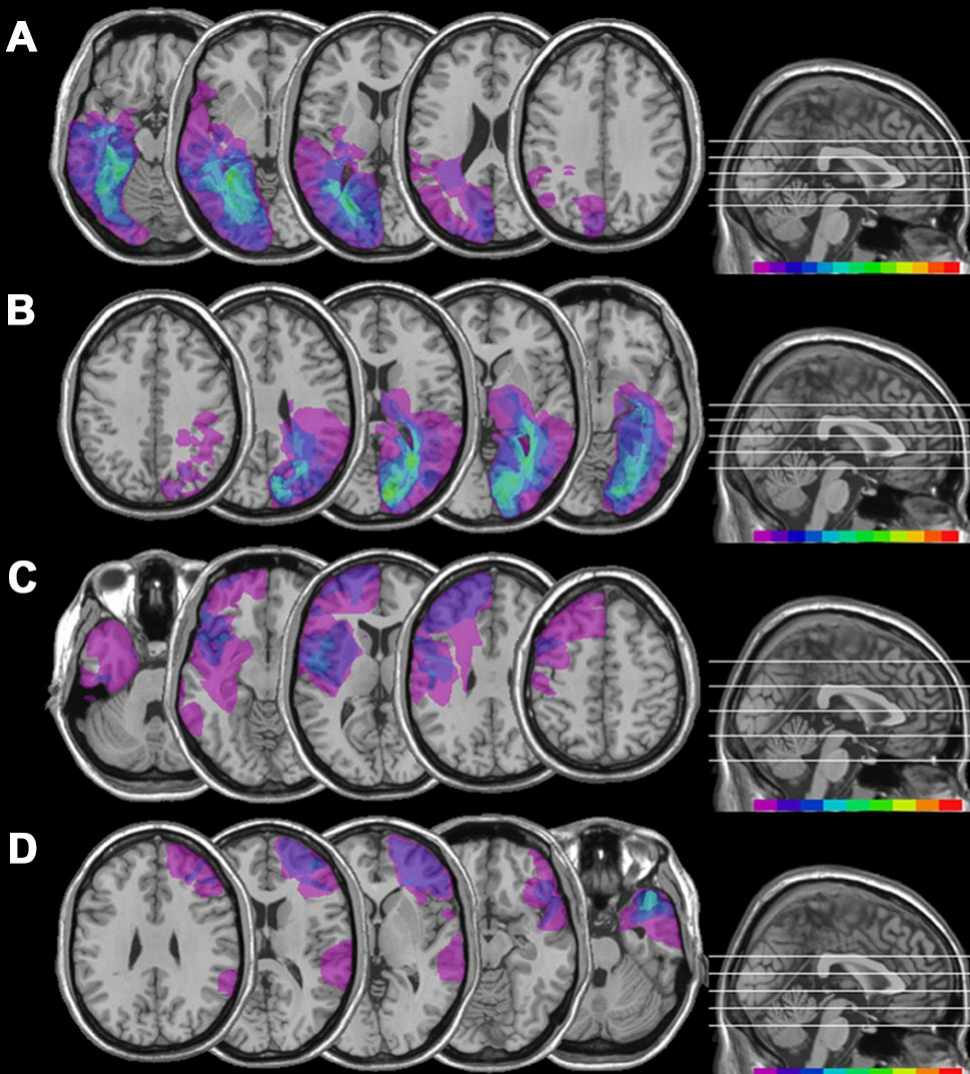
Location and overlap of brain lesions of patients. The image shows the lesions of the hemianopic patients with left posterior brain lesion (**A**), hemianopic patients with right posterior brain lesion (**B**), control patients with left anterior brain lesion (**C**), control patients with right anterior brain lesion (**D**)**,** projected onto four axial slices of the standard MNI brain. The levels of the axial slices are marked by white lines on the sagittal view of the brain. The color bar indicates the number of overlapping lesions

Mapping of brain lesions was performed using MRIcro. Lesions documented by the most recent clinical CT or MRI were traced onto the T1-weighted MRI template from the Montreal Neurological Institute provided with MRIcro software (Rorden et al. 2007; Rorden and Brett 2000), with the exception of HEMI L-LES 7 and HEMI R-LES 8 whose MRI/CT scans were not available. Although lesion reconstruction was not performed for these two patients, radiology MRI/CT reports were available and confirmed the presence of unilateral lesions limited to the posterior cortices. Lesion volumes were computed for each patient in order to compare the extension of the lesions among the four patients’ groups. No significant differences (one-way ANOVA, *F*_1,39_ = 1.61; *p* = 0.201) in lesion volumes between the four groups of patients were found (see Fig. 1). Patients with posterior lesions were recruited based on reported visual field defects, the availability of a visual field perimetry and CT/MRI reports of the lesion. In patients with posterior right lesions, the presence of neglect was screened using the Behavioral Inattention Test (Wilson et al. 1987), to ensure performance was in the normal range.

All patients showed normal or corrected-to-normal visual acuity. Patients were informed about the procedure and the purpose of the study and gave written informed consent. The study was designed and performed in accordance with the ethical principles of the Declaration of Helsinki and was approved by the Ethics Committee of the Regional Health Service Romagna (CEROM; n.2300).

### EEG recordings

Participants comfortably seated in a soundproof room, while EEG signal was recorded from five sessions of one minute each, in an eyes-closed resting state condition. EEG data were acquired through a BrainAmp DC amplifier (BrainProducts GmbH, Germany) and Ag/AgCl electrodes (Fast’nEasy Cap, Easycap GmbH, Germany) from 59 scalp sites (Fp1, AF3, AF7, F1, F3, F7, FC1, FC3, FC5, FT7, C1, C3, C5, T7, CP1, CP3, CP5, TP7, P1, P3, P5, P7, PO3, PO7, O1, Fp2, AF4, AF8, F2, F4, F8, FC2, FC4, FC6, FT8, C2, C4, C6, T8, CP2, CP4, CP6, TP8, P2, P4, P6, P8, PO4, PO8, O2, FPz, AFz, Fz, FCz, Cz, CPz, Pz, POz, Oz) and the right mastoid. As online reference, the left mastoid electrode was used, while the ground electrode was placed on the right cheek. Vertical and horizontal electrooculogram (EOG) components were recorded from above and below the left eye, and from the outer canthus of each eye. Data were recorded with a band-pass filter of 0.01–100 Hz and digitized at a sampling rate of 1000 Hz, while impendences were kept under 5 KΩ.

### EEG preprocessing

EEG recordings were processed off-line using EEGlab (v14.1.2; Delorme and Makeig 2004) and custom scripts developed in Matlab (R2018a; The Mathworks Inc., USA). Data from all electrodes were re-referenced to the average of all scalp electrodes and filtered with a band-pass filter of 1–100 Hz. Continuous signals were segmented in epochs of 1 s. Horizontal and vertical eye artifacts were visually identified and corrected with an independent component analysis (ICA), after data dimension reduction by means of Principal Component Analysis (PCA). Data were down-sampled to 250 Hz and current source density (CSD) interpolation, using spherical splines (Kayser and Tenke 2015) was applied to minimize confounding effects in inter-electrode synchronization due to volume conduction and field spread (van Diessen et al. 2015; Cohen 2014). CSD transformation was performed in Matlab using the open-source CSD toolbox (version 1.1; http://psychophysiology.cpmc.columbia.edu/Software/CSDtoolbox/).

### Functional connectivity analysis

A multi-step approach was used to investigate, separately for each frequency band and hemisphere, the effects of unilateral posterior or anterior brain lesions on functional connectivity and resting-state network topology.

#### Weighted phase-lag index

Functional connectivity was measured computing the weighted phase-lag index (wPLI, Vinck et al. 2011), which extends the phase-lag index (PLI, Stam et al. 2007) by weighting the contribution of observed phase leads and lags by the magnitude of the imaginary component of the cross-spectrum (Vinck et al. 2011). The wPLI is based on a consistent lag between the instantaneous phases of two electrodes and is less sensitive to zero-lag phase-relations typical for common sources (Bastos and Schoffelen 2016; Hardmeier et al. 2014).

To compute the wPLI, frequency decomposition was performed for all EEG channels, using a multitaper method with digital prolate spheroidal sequence (DPSS) windows, implemented in Fieldtrip toolbox (v20210311) for EEG/MEG-analysis (for the details of the implementations, see Vinck et al. 2011).

Complex Fourier coefficients were extracted for the frequency bands of interest, specifically in the theta (3–6 Hz), low alpha (7–10 Hz), upper alpha (11–13 Hz) and beta (14–25 Hz) ranges and 59 × 59 connectivity matrix was constructed for each participant and frequency band of interest. Then, the wPLIs computed for all possible pairs of electrodes within the left (Fp1, AF3, AF7, F1, F3, F7, FC1, FC3, FC5, FT7, C1, C3, C5, T7, CP1, CP3, CP5, TP7, P1, P3, P5, P7, PO3, PO7, O1) and the right (Fp2, AF4, AF8, F2, F4, F8, FC2, FC4, FC6, FT8, C2, C4, C6, T8, CP2, CP4, CP6, TP8, P2, P4, P6, P8, PO4, PO8, O2) hemisphere were averaged across pairs of electrodes and participants, to obtain an index of intrahemispheric functional connectivity. Electrodes on the sagittal midline were excluded from the analysis, to provide a better segregation of the signal of the two hemispheres. Finally, the wPLIs computed for all possible pairs of electrodes between the left and the right hemisphere were averaged across pairs of electrodes and participants, to obtain an index of interhemispheric functional connectivity. Electrodes on the sagittal midline were excluded from the analysis, to provide a better segregation of the signal between the left and the right hemisphere.

Intrahemispheric functional connectivity was compared among groups and hemispheres, separately for each frequency band (i.e., theta, lower alpha, upper alpha, beta), with an ANOVA on mean intrahemispheric wPLI having *Group* (Healthy participants, Left-lesioned hemianopic patients, Right-lesioned hemianopic patients, Left-lesioned control patients, Right-lesioned control patients) as between-subjects factor, and *Hemisphere* (Left, Right) as within-subjects factor. Last, to test for differences among groups in interhemispheric functional connectivity separately for each frequency band (i.e. theta, lower alpha, upper alpha, beta), an ANOVA on mean interhemispheric wPLI having *Group* (Healthy participants, Left-lesioned hemianopic patients, Right-lesioned hemianopic patients, Left-lesioned control patients, Right-lesioned control patients) as between-subjects factor was run. Statistically significant interactions or main effects were subsequently explored through simple planned contrasts, comparing connectivity in each group of patients against connectivity in the group of Healthy participants.

#### Graph theory

Two graph theory measures, the clustering coefficient (C) and the characteristic path length (L), were chosen to assess the functional network segregation and integration, respectively. In particular, the C represents the degree to which nodes in a graph are interconnected whereas the L reflects the average shortest path length between all pairs of nodes in the network (Watts and Strogatz 1998). Graph indices were computed with custom scripts developed in Matlab, through the Brain Connectivity Toolbox (v1.1, Rubinov and Sporns 2010). Specifically, the C and the L were calculated separately for each band of interest (i.e., theta, low alpha, upper alpha, beta), on undirected weighted network matrices without thresholding, putting wPLI values as the edge weights.

The C (Onnela, et al. 2005) was defined as:

$$C^{w} = \frac{1}{n} \mathop \sum \limits_{i \in N} \frac{{2t_{i}^{w} }}{{k_{i} \left( {k_{i} - 1} \right)}}$$

where C is the clustering coefficient of a given node (for details, see Rubinov and Sporns 2010).

The L (Runinov and Sporns, 2010) was defined as:

$$L^{w} = \frac{1}{n} \mathop \sum \limits_{i \in N} \frac{{\mathop \sum \nolimits_{j \in N,j \ne i} d_{ij}^{w} }}{n - 1}$$

where L is the average distance between a given node and all other nodes (for details, see Rubinov and Sporns 2010).

To test for differences in the functional integration and segregation within the two brain hemispheres, the C and the L parameters were separately computed for the left and the right hemisphere and averaged across participants. Electrodes on the sagittal midline were excluded from the analysis, to provide a better segregation of the signal of the two hemispheres. Then, possible differences among groups and hemispheres were tested separately for each frequency band (i.e. theta, low alpha, upper alpha, beta) with an ANOVA having *Group* (Healthy participants, Left-lesioned hemianopic patients, Right-lesioned hemianopic patients, Left-lesioned control patients, Right-lesioned control patients) as between-subjects factor, and *Hemisphere* (Left, Right) as within-subjects factor. Statistically significant main effects or interactions were subsequently explored through simple planned contrasts**,** by comparing the mean C and L of each group of patients against the group of Healthy participants.

### Computerized visual field test

In addition to the EEG recording, hemianopic patients’ visual detection abilities were also tested (Làdavas et al. 2020; Grasso et al. 2016; Dundon et al. 2015a, b; Passamonti et al. 2009; Bolognini et al. 2005) during the clinical examination, to explore a possible link between visual performance and functional connectivity. Patients sat at a viewing distance of 120 cm, while a stimulus array of 52° × 45° (horizontally and vertically, respectively) was projected on the wall. Targets, consisting of white dots (1°) were presented on a black background for 100 ms, at random positions. A red fixation cross (0.5°) was displayed on the center of the screen. A total of 96 targets was presented (i.e., 48 targets for each hemifield). In 31 trials, no target was presented (i.e., catch trials). Patients were instructed to press a response button after the detection of the target. Patients’ gaze was monitored throughout the task by the experimenter. The task was performed in two different conditions: when patients were not allowed to move their eyes to compensate for the visual field loss and had to keep their gaze on a central fixation cross (Fixed-eyes) and when patients were allowed to perform eye movements (Eye movements). Visual detections and false alarms rates for stimuli presented in the blind visual field were measured. D prime (perceptual sensitivity) was calculated and used for subsequent correlational statistical analysis with the patients’ wPLI and Graph theory indices that resulted to be impaired, compared to healthy participants.

## Results

### Functional connectivity in the theta band

#### Intrahemispheric wPLI

**Fig. 2:**
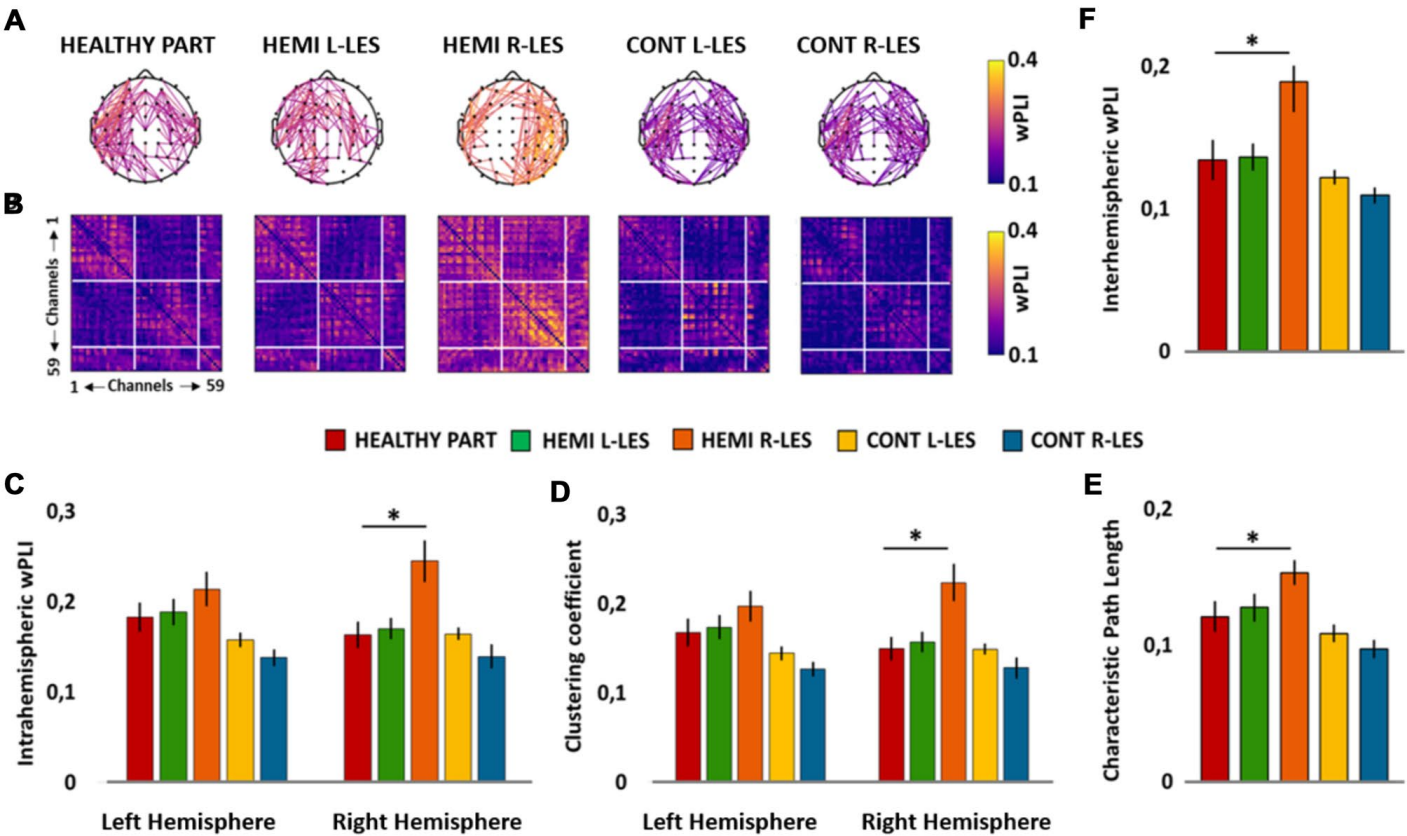
Scalp maps **A** represent the strongest theta connections (*n* = 171, 10% of total connections) in the group of healthy participants (HEALTHY PART), hemianopic patients with left lesion (HEMI L-LES), hemianopic patients with right lesion (HEMI R-LES), control patients with left anterior lesion (CONT L-LES) and control patients with right anterior lesion (CONT R-LES) The color bar represents the wPLI value, so that higher values are associated with yellow color and lower values with blue color. Panel **B** represents the theta band functional connectivity matrices of the wPLIs calculated for each of the five groups. Bar histograms show the mean theta (3–6 Hz) intrahemispheric wPLI relative to the left and to the right hemisphere, within each group (**C**), the mean theta clustering coefficient relative to the left and to the right hemisphere, within each group (**D**)**,** the mean theta characteristic path length within each group (**E**) and the mean theta interhemispheric wPLI within each group (**F**)**.** Error bars represent standard error; asterisks indicate the significant comparisons

The ANOVA showed a significant main effect of *Group* (*F*_4,54_ = 5.185, *p* = 0.001, *η*_*p*_^2^ = 0.277), whereas the main effect of *Hemisphere* (*F*_1,54_ = 0.001, *p* = 0.977, *η*_*p*_^2^ < 0.001) was not significant. Importantly, the ANOVA showed also a significant *Group* × *Hemisphere* (*F*_4,54_ = 2.879, *p* = 0.031, *η*_*p*_^2^ = 0.176; see Fig. 2C) interaction, which was further explored through simple planned contrasts. More specifically, the intrahemispheric theta wPLI of each group of patients was contrasted against the group of healthy participants, separately for the left and the right hemisphere.

Planned contrast performed on the group of Left-lesioned hemianopics *vs* Healthy participants revealed no significant difference between the two groups in the left hemisphere (Left-lesioned hemianopics *η*_*p*_^2^ 0.19, Healthy participants *M* = 0.18; *p* = 0.776) nor in the right hemisphere (Left-lesioned hemianopics *M* = 0.17, Healthy participants *M* = 0.16; *p* = 0.743). Similarly, planned contrast on the group of Right-lesioned hemianopics vs Healthy participants did not show a significant difference in the left hemisphere (Right-lesioned hemianopics *M* = 0.21; *p* = 0.141). However, Right-lesioned hemianopics exhibited a significantly increased wPLI in the right hemisphere (*M* = 0.25), compared to the right hemisphere of healthy participants (*p* < 0.001). Last, planned contrast on the group of both Right-lesioned and Left-lesioned control patients *vs* Healthy participants revealed no significant difference neither in the left (all ps > 0.057) or in the right (all ps > 0.314) hemisphere.

#### Clustering coefficient

The ANOVA revealed a significant main effect of *Group* (*F*_4,54_ = 5.249, *p* = 0.001, *η*_*p*_^2^ = 0.280), whereas the main effect of *Hemisphere* (*F*_1,54_ = 0.005, *p* = 0.943, *η*_*p*_^2^ < 0.001) was not significant. Importantly, the ANOVA showed also a significant *Group* × *Hemisphere* (*F*_4,54_ = 2.591, *p* = 0.047, *η*_*p*_^2^ = 0.161; see Fig. 2D) interaction, which was further explored by performing planned contrast.

Planned contrast performed on the group of Left-lesioned hemianopics against the group of Healthy participants did not show any significant difference neither in the left (Left-lesioned hemianopics *M* = 0.17, Healthy participants *M *= 0.17, *p* = 0.754) or in the right (Left-lesioned hemianopics *M* = 0.16, Healthy participants *M* = 0.17, *p* = 0.698) hemisphere. Planned contrast on the group of Right-lesioned hemianopics *vs* Healthy participants revealed no significant difference in the left hemisphere (Right-lesioned hemianopics *M* = 0.20, *p* = 0.132) but, importantly, Right-lesioned hemianopics exhibited a higher theta C in the right hemisphere, compared to the right hemisphere of Healthy participants (Right-lesioned hemianopics *M* = 0.22, *p* < 0.001).

Last, for both groups of Left-lesioned and Right-lesioned control patients, planned contrast performed against Healthy participants showed no significant difference in the left hemisphere (all ps > 0.057) nor in the right hemisphere (all ps > 0.316).

#### Characteristic path length

The ANOVA showed no significant main effect of *Hemisphere* (*F*_1,54_ = 0.657, *p* = 0.421, *η*_*p*_^2^ = 0.012), nor a significant *Group* × *Hemisphere* interaction (*F*_4,54_ = 1.036, *p* = 0.397, *η*_*p*_^2^ = 0.071), but a significant main effect of *Group* (*F*_4,54_ = 4.445, *p* = 0.004, *η*_*p*_^2^ = 0.248; see Fig. 2E).

Importantly, planned contrast revealed a significantly greater theta L for the group of Right-lesioned hemianopics (*M* = 0.15) compared to Healthy participants (*M* = 0.12, *p* = 0.015), whereas no other significant difference was found (all ps > 0.106).

#### Interhemispheric wPLI

The ANOVA on the interhemispheric wPLI in the theta band showed a significant main effect of *Group* (*F*_4,54_ = 4.638, *p* = 0.003, *η*_*p*_^2^ = 0.256; see Fig. 2F). Planned contrasts comparing the theta interhemispheric wPLI of each group of patients against the group of healthy controls revealed a significantly higher interhemispheric wPLI for the group of Right-lesioned hemianopics (*M* = 0.19), compared to healthy participants (*p* = 0.005). No other significant difference was found (all ps > 0.235).

### Functional connectivity in the lower alpha band

#### Intrahemispheric wPLI

**Fig. 3:**
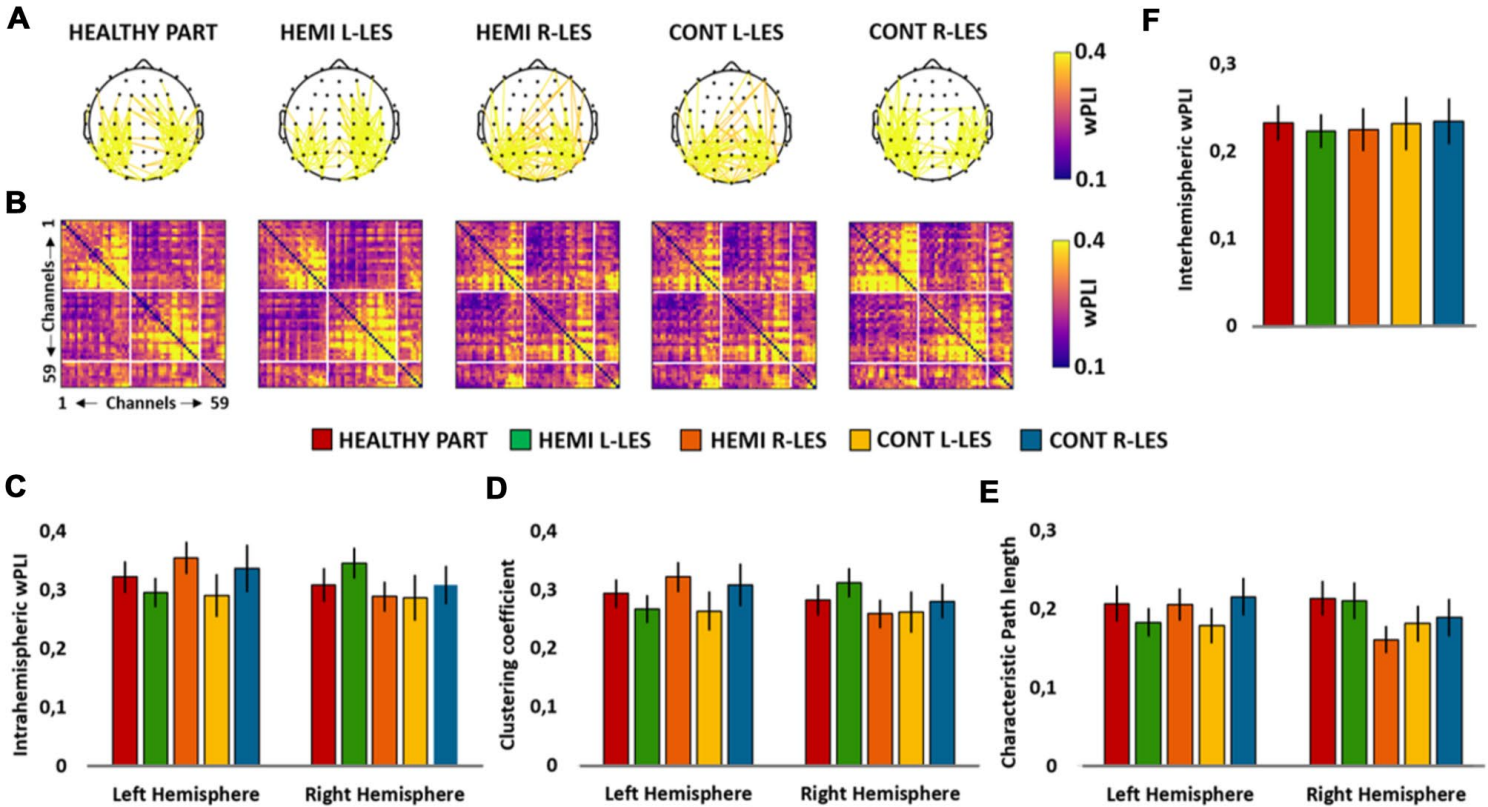
Scalp maps **A** represent the strongest lower alpha connections (*n* = 171, 10% of total connections) in the group of healthy participants (HEALTHY PART), hemianopic patients with left lesion (HEMI L-LES), hemianopic patients with right lesion (HEMI R-LES), control patients with left anterior lesion (CONT L-LES) and control patients with right anterior lesion (CONT R-LES) The color bar represents the wPLI value, so that higher values are associated with yellow color and lower values with blue color. Panel **B** represents the lower alpha band functional connectivity matrices of the wPLIs calculated for each of the five groups. Bar histograms show the mean lower alpha (7–10 Hz) intrahemispheric wPLI relative to the left and to the right hemisphere, within each group (**C**), the mean lower alpha clustering coefficient relative to the left and to the right hemisphere, within each group (**D**), the mean lower alpha characteristic path length relative to the left and to the right hemisphere, within each group (**E**), the mean lower alpha interhemispheric wPLI within each group (**F**). Error bars represent standard error; asterisks indicate the significant comparisons

The ANOVA on the intrahemispheric wPLI in the lower alpha band did not show a significant main effect of *Group* (*F*_4,54_ = 0.226, *p* = 0.923, *η*_*p*_^2^ = 0.016), nor a significant effect of *Hemisphere* (F_1,54_ = 1.134, *p* = 0.292, *η*_*p*_^2^ = 0.021), but a significant *Group* × *Hemisphere* (*F*_4,54_ = 3.162, *p* = 0.021, *η*_*p*_^2^ = 0.190; see Fig. 3C) interaction. However, planned contrast performed on this significant interaction showed no significant differences for none of the groups of patients, compared to healthy participants, neither in the left (all ps > 0.408) nor in the right hemisphere (all ps > 0.349).

#### Clustering coefficient

The ANOVA on the low alpha C did not reveal a significant main effect of *Group* (*F*_4,54_ = 0.204, *p* = 0.935, *η*_*p*_^2^ = 0.015), nor a significant main effect of *Hemisphere* (*F*_1,54_ = 1.247, *p* = 0.269, *η*_*p*_^2^ = 0.023), but a significant *Group* × *Hemisphere* (*F*_4,54_ = 3.266, *p* = 0.019, *η*_*p*_^2^ = 0.193; see Fig. 3D) interaction.

However, planned contrast showed no significant differences for none of the groups of patients, compared to healthy participants, neither in the left (all ps > 0.443) or in the right hemisphere (all ps > 0.416).

#### Characteristic path length

The ANOVA revealed no significant main effect of *Group* (*F*_4,54_ = 0.436, *p* = 0.782, *η*_*p*_^2^ = 0.031) and *Hemisphere* (*F*_1,54_ = 0.493, *p* = 0.486, *η*_*p*_^2^ = 0.009), nor a significant *Group* × *Hemisphere* interaction (*F*_4,54_ = 1.903, *p* = 0.123, *η*_*p*_^2^ = 0.124; see Fig. 3E).

#### Interhemispheric wPLI

Last, the ANOVA performed on the interhemispheric wPLI in the low alpha band did not reveal a significant main effect of *Group* (*F*_4,54_ = 0.048, *p* = 0.996, *η*_*p*_^2^ = 0.004; see Fig. 3F).

### Functional connectivity in the upper alpha band

#### Intrahemispheric wPLI

**Fig. 4:**
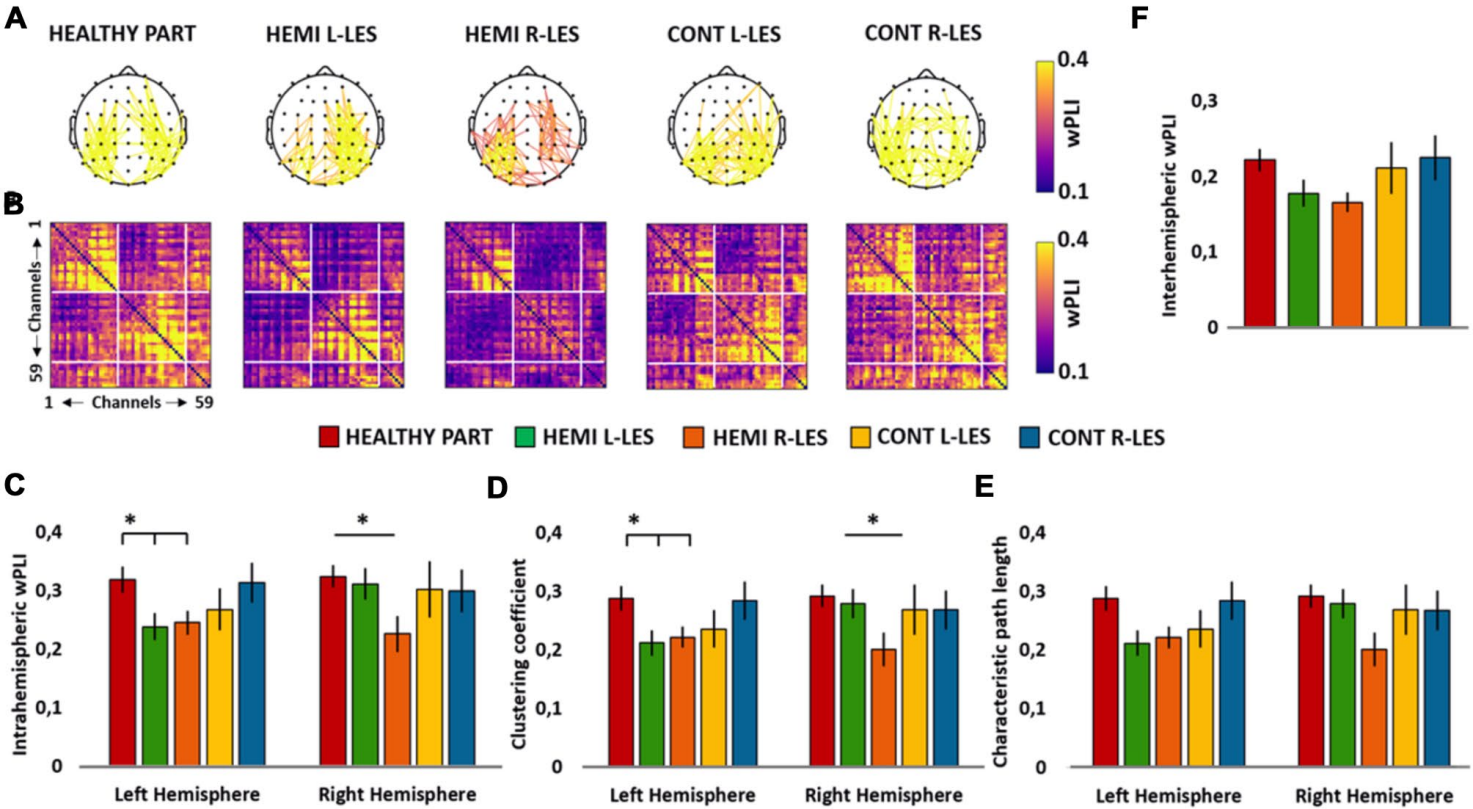
Scalp maps **A** represent the strongest upper alpha connections (*n* = 171, 10% of total connections) in the group of healthy participants (HEALTHY PART) hemianopic patients with left lesion (HEMI L-LES), hemianopic patients with right lesion (HEMI R-LES), control patients with left anterior lesion (CONT L-LES) and control patients with right anterior lesion (CONT R-LES) The color bar represents the wPLI value, so that higher values are associated with yellow color and lower values with blue color. Panel **B** represents the upper alpha band functional connectivity matrices of the wPLIs calculated for each of the five groups. Bar histograms show the mean upper alpha (11–13 Hz) intrahemispheric wPLI relative to the left and to the right hemisphere, within each group (**C**), the mean upper alpha clustering coefficient relative to the left and to the right hemisphere, within each group (**D**), the mean upper alpha characteristic path length relative to the left and to the right hemisphere, within each group (**E**) and the mean upper alpha interhemispheric wPLI within each group **(F**). Error bars represent standard error; asterisks indicate the significant comparisons

For the upper alpha band, the ANOVA on the intrahemispheric wPLI did not reveal a significant main effect of *Group* (*F*_4,54_ = 1.791, *p* = 0.144, *η*_*p*_^2^ = 0.017), nor a significant main effect of *Hemisphere* (*F*_1,54_ = 2.129, *p* = 0.150, *η*_*p*_^2^ = 0.038) but, importantly, the ANOVA showed a significant *Group* × *Hemisphere* (*F*_4,54_ = 2.787, *p* = 0.035, *η*_*p*_^2^ = 0.171; see Fig. 4C) interaction.

Planned contrast performed on the group of Left-lesioned hemianopics *vs* Healthy participants revealed a significantly decreased wPLI in the left hemisphere of Left-lesioned hemianopics (*M* = 0.24), compared to the left hemisphere of Healthy participant (*M* = 0.32, *p* = 0.020), whereas no significant difference between the two groups was found in the right hemisphere (Left-lesioned hemianopics *M* = 0.31, Healthy participants *M* = 0.32, *p* = 0.749). For the group of Right-lesioned hemianopics, planned contrasts revealed a significant decrease in wPLI both in the left (*M* = 0.25; *p* = 0.036) and in the right (*M* = 0.22; *p* = 0.019) hemisphere, compared to healthy participants. Last, for both groups of Left-lesioned and Right-lesioned control patients, planned contrast against Healthy participants revealed no significant difference neither in the left (all ps > 0.186), nor in the right hemisphere (all ps > 0.582).

#### Clustering coefficient

The ANOVA on the C in the upper alpha band did not show a significant main effect of *Group* (*F*_4,54_ = 1.749, *p* = 0.153, *η*_*p*_^2^ = 0.115), nor a significant main effect of *Hemisphere* (F_1,54_ = 1.757, *p* = 0.191, *η*_*p*_^2^ = 0.032), but a significant *Group* × *Hemisphere* (*F*_4,54_ = 2.958, *p* = 0.028, *η*_*p*_^2^ = 0.180, see Fig. 4D) interaction.

Planned contrast on Left-lesioned hemianopics *vs* Healthy participants revealed a significantly lower C in the left hemisphere of Left-lesioned hemianopics (*M* = 0.21), compared to the left hemisphere of Healthy participants (*M* = 0.29, *p* = 0.017), whereas no significant difference between the two groups was found for the right hemisphere (Left-lesioned hemianopics *M* = 0.28, Healthy participants *M* = 0.29; *p* = 0.727). Similarly, also for the group of Right-lesioned hemianopics, planned contrasted revealed a significantly lower C in the left hemisphere (*M* = 0.22), compared to the left hemisphere of healthy participants (*p* = 0.041). Furthermore, Right-lesioned hemianopics exhibited also a lower C in the right hemisphere (*M* = 0.20), compared to the right hemisphere of Healthy participants (*p* = 0.020). In contrast, for both groups of Left-lesioned and Right-lesioned control patients, no significant comparison was found in the left hemisphere (all ps > 0.142), nor in the right hemisphere (all ps > 0.573), relative to Healthy participants.

#### Characteristic path length

The ANOVA revealed no significant main effect of *Group* (*F*_4,54_ = 2.210, *p* = 0.080, *η*_*p*_^2^ = 0.141) and *Hemisphere* (*F*_1,54_ = 0.486, *p* = 0.489, *η*_*p*_^2^ = 0.09), nor a significant *Group* × *Hemisphere* interaction (*F*_4,54_ = 2.079, *p* = 0.096, *η*_*p*_^2^ = 0.133; see Fig. 4E).

#### Interhemispheric wPLI

Last, the ANOVA performed on the upper alpha interhemispheric wPLI did not show a significant main effect of *Group* (*F*_4,54_ = 1.748, *p* = 0.153, *η*_*p*_^2^ = 0.115, see Fig. 4F).

### Functional connectivity in the beta band

#### Intrahemispheric wPLI

**Fig. 5:**
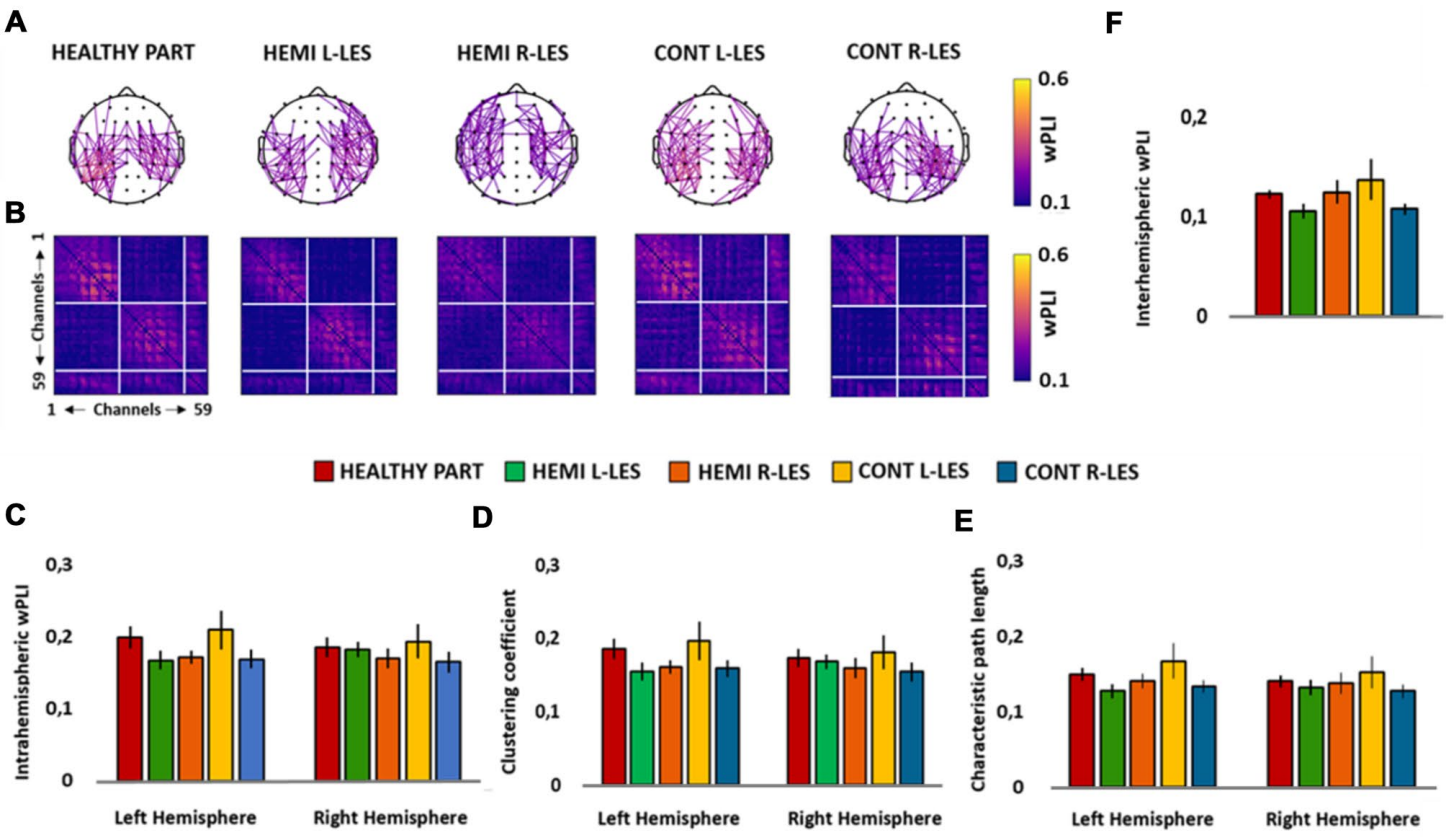
Scalp maps **A** represent the strongest beta connections (*n* = 171, 10% of total connections) in the group of healthy participants (HEALTHY PART) hemianopic patients with left lesion (HEMI L-LES), hemianopic patients with right lesion (HEMI R-LES), control patients with left anterior lesion (CONT L-LES) and control patients with right anterior lesion (CONT R-LES) The color bar represents the wPLI value, so that higher values are associated with yellow color and lower values with blue color. Panel **B** represents the beta band functional connectivity matrices of the wPLIs calculated for each of the five groups. Bar histograms show the mean beta (14–25 Hz) intrahemispheric wPLI relative to the left and to the right hemisphere, within each group **(C)**, the mean beta clustering coefficient relative to the left and to the right hemisphere, within each group **(D)**, the mean beta Characteristic path length relative to the left and to the right hemisphere, within each group **(E)** and the mean beta interhemispheric wPLI within each group **(F)**. Error bars represent standard error; asterisks indicate the significant comparisons

The ANOVA performed on the intrahemispheric wPLI in the beta band revealed no significant main effect of *Group* (*F*_4,54_ = 0.984, *p* = 0.423, *η*_*p*_^2^ = 0.105), nor a significant main effect of *Hemisphere* (*F*_1,54_ = 0.593, *p* = 0.444, *η*_*p*_^2^ = 0.053). In addition, the ANOVA showed no significant *Group* × *Hemisphere* (*F*_4,54_ = 1.180, *p* = 0.329, *η*_*p*_^2^ = 0.100; see Fig. 5C) interaction.

#### Clustering coefficient

The ANOVA performed on the C in the beta band showed no significant main effect of *Group* (*F*_4,54_ = 0.979, *p* = 0.426, *η*_*p*_^2^ = 0.108), or *Hemisphere* (*F*_1,54_ = 0.593, *p* = 0.444, *η*_*p*_^2^ = 0.032), nor a significant *Group* × *Hemisphere* (*F*_4,54_ = 2.958, *p* = 0.128, *η*_*p*_^2^ = 0.180, see Fig. 5D) interaction.

#### Characteristic path length

The ANOVA did not reveal a significant main effect of *Group* (*F*_4,54_ = 1.074, *p* = 0.378 0, *η*_*p*_^2^ = 0.112) and *Hemisphere* (*F*_1,54_ = 0.590, *p* = 0.212, *η*_*p*_^2^ = 0.105). Also the *Group* × *Hemisphere* interaction (F_4,54_ = 1.645, *p* = 0.622, *η*_*p*_^2^ = 0.009; see Fig. 5E) was not significant.

#### Interhemispheric wPLI

Also, the ANOVA on the beta interhemispheric wPLI showed no significant main effect of *Group* (*F*_4,54_ = 1.252, *p* = 0.298, *η*_*p*_^2^ = 0.088, see Fig. 5F).

### Hemianopic patients’ visual performance and functional connectivity

Finally, we tested whether altered theta and upper alpha functional connectivity can relate to behavioral performance in visual detection tests in hemianopic patients with both left and right lesions. To this aim, the relationship between hemianopic patients' perceptual sensitivity (D prime) in their blind field at the Computerized Visual Field test and their functional connectivity parameters that resulted to be impaired was explored. More specifically, hemianopic patients’ D prime in both the Fixed-Eyes condition and in the Eye-Movements condition was correlated to the intrahemispheric wPLI, the C and the L in the theta band and to the intrahemispheric wPLI and the C in the upper alpha band, separately for the lesioned and the intact hemisphere and, last, to the interhemispheric wPLI in the theta band. Pearson correlations were performed (Bonferroni-Holm corrections were used for multiple comparisons; adjusted p-levels are reported).

In the Fixed-Eyes condition, no significant correlation between the theta intrahemispheric wPLI in both the lesioned and the intact hemisphere and the D prime was found (all ps = 0.100). Similarly, no significant correlation was found between visual performance and the theta C in both the lesioned and the intact hemisphere (all ps = 0.100) and visual performance and the theta L in the lesioned or the intact hemisphere (all ps = 0.100). Last, no significant correlation between interhemispheric theta wPLI and D prime in the blind visual field was found (*r* = – 0.351, *p* = 0.072), therefore, suggesting no relationship between connectivity parameter in the theta range and visual detection performance in the blind visual field when eye movements were prevented.

**Fig. 6:**
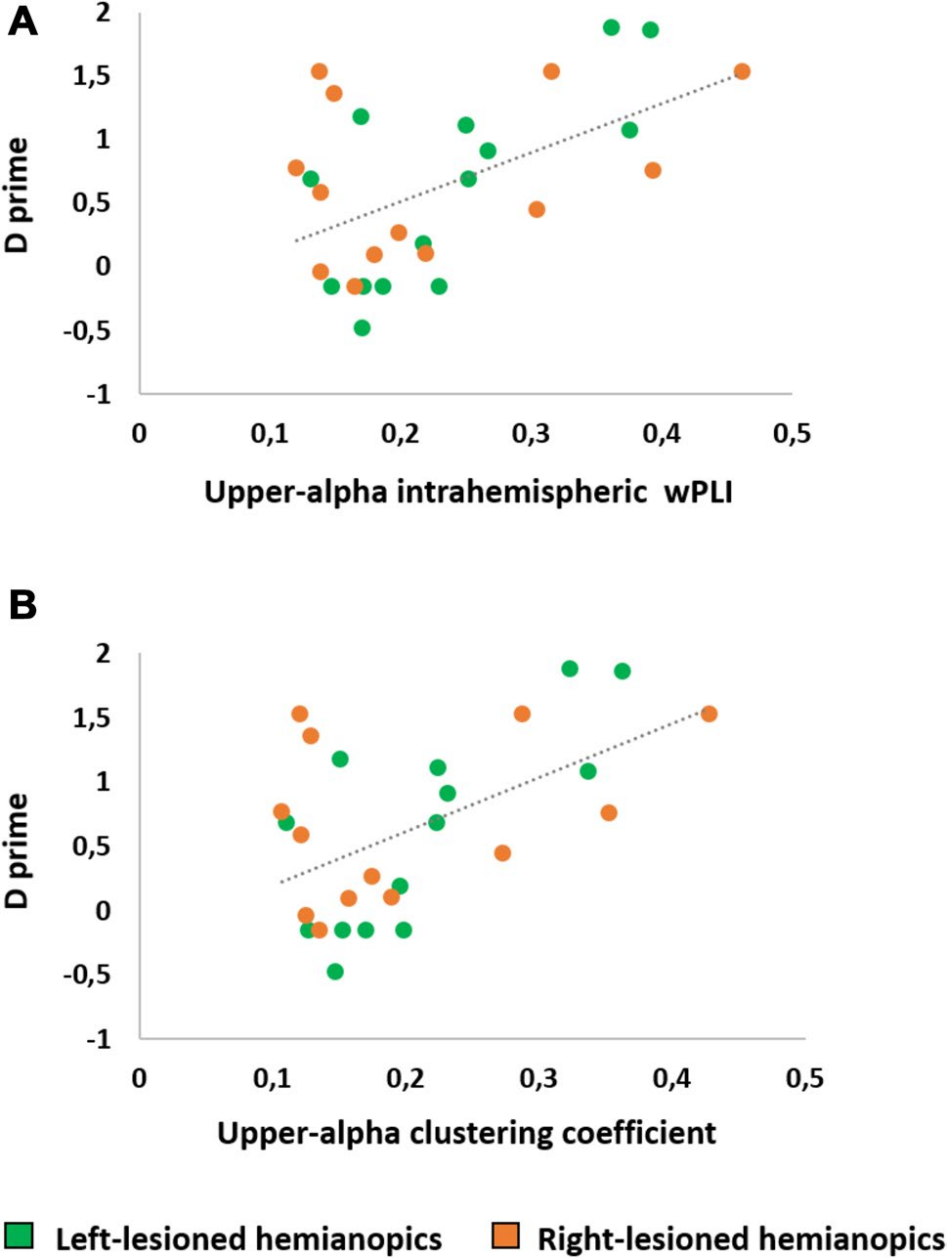
Correlations between the hemianopic patients' visual performance in the Computerized Visual Field test in the Fixed-Eyes condition and functional connectivity parameters. Panel **A** depicts the correlation between D prime and upper alpha intrahemispheric wPLI; Panel **B** depicts the correlation between D prime and the upper alpha clustering coefficient

As for the upper alpha range, no significant correlation between the intrahemispheric wPLI in the intact hemisphere and the D prime (*r* = 0.225, *p* = 0.257) was found, but, importantly, a positive significant correlation between the upper alpha intrahemispheric wPLI in the lesioned hemisphere and the D prime (*r* = 0.496, *p* = 0.016, see Fig. 6A) emerged (i.e., the higher the upper alpha intrahemispheric wPLI in the lesioned hemisphere, the better the performance in patients’ blind field). In line, also a positive significant correlation between upper alpha C in the lesioned hemisphere and the D prime (*r* = 0.541, *p* = 0.006, see Fig. 6B) was found, suggesting that higher upper alpha C in the lesioned hemisphere is predictive of better performance in hemianopic patients’ blind field. In contrast, no significant correlation between the upper alpha C in the intact hemisphere and the D prime was found (*r* = 0.264, *p* = 0.182). Overall, these findings indicate that upper alpha functional connectivity in the lesioned hemisphere in hemianopic patients is associated with visual detection performance in their blind visual field.

Finally, in the Eyes-Movements condition, no significant correlation with hemianopic patients’ D prime in the blind field was found (all ps > 0.074), suggesting that both the theta and the upper alpha functional connectivity are not related to patients’ ability to compensate for the field loss when eye movements are allowed.

## Discussion

The present study has revealed that posterior brain lesions selectively impair oscillatory brain connectivity at rest. More precisely, both hemianopics with left and right posterior lesions showed a reduction in intrahemispheric functional connectivity in the range of upper alpha (11–13 Hz) during resting state, while control patients with anterior lesions revealed no connectivity change compared to healthy individuals. Moreover, posterior right lesions seemed to induce more severe alterations in the oscillatory connectivity patterns, with hemianopics with right lesions showing a reduced intrahemispheric upper alpha connectivity in both the lesioned and the intact hemisphere and additional abnormal increase in connectivity in the theta range. On the contrary, left-lesioned hemianopics revealed only a reduction in intrahemispheric upper alpha connectivity limited to the lesioned hemisphere.

These results are in line with previous literature documenting dysfunctions in connectivity patterns after brain damage (Dubovik et al. 2012; Westlake et al. 2012; Wu et al. 2011; Castellanos et al. 2010; Guiggisberg et al. 2008) but also add to previous knowledge that lesions to posterior cortices, damaging the crucial hub for the generation and distribution of alpha oscillations, specifically impair connectivity in this frequency band. Accordingly, previous evidence on hemianopics revealed that posterior lesions impair oscillations in the alpha range, with a slowdown of the speed of processing in alpha (i.e., a reduction of alpha peak), the occurrence of an interhemispheric power imbalance, in favor of the intact hemisphere (Pietrelli et al. 2019) and a reduction of alpha reactivity induced by eyes-opening (Gallina et al. 2021). Therefore, as a whole, these findings corroborate the notion that alpha oscillatory patterns at rest can reflect the underlying functioning of the visual system, since lesions to posterior visual brain regions specifically impair activity in this frequency band. In line with this view, alpha oscillations represent the dominant frequency range of activity of the resting human brain and their distribution is prominent over posterior cerebral regions (Rosanova et al. 2009; Berger 1929). Accordingly, posterior visual cortices have been demonstrated to be crucial in generating and propagating alpha oscillations in the visual system (Hindriks et al. 2015; Bollimunta et al. 2008). In addition, oscillations in the alpha range are reportedly linked to visual perception and visual cortex activity (Romei et al. 2008a, 2008b; Pfurtscheller et al. 1994).

Hemianopic patients with posterior lesions showed connectivity impairments only in the upper range of the alpha band, consistently with previous evidence reporting, in these patients, a shift towards slower frequencies of the individual alpha peak (Pietrelli et al. 2019). Indeed, converging evidence have reported that occipito-parietal alpha generators, which might be impaired after posterior lesions, are more linked with oscillatory activity in the upper alpha band (Cantero et al. 2002). In line, the activity of the parieto-occipital oscillations in the upper alpha range has been consistently linked with a greater efficiency of the visual processing, with the faster alpha frequencies being related to a more effective task execution both within (Zhang et al. 2019; Wutz et al. 2018; Minami and Amano 2017; Samaha and Postle 2015) and across (Venskus and Huighes 2021; Cooke et al. 2019; Cecere et al. 2015) sensory modalities.

Importantly, hemianopic patients with posterior lesions did not show altered patterns of functional connectivity in the beta range at rest, both intra- and inter-hemispheres. Altered oscillatory activity in the beta band has been reported in stroke patients in task-related oscillatory activity (Guggisberg et al. 2015) and also in hemianopics, mostly associated to impaired stimulus processing in visual and attentional tasks (Allaman et al. 2021; Sanchez-Lopez et al. 2020). However, the present findings suggest that, when looking at oscillatory patterns at rest, a damage to the posterior brain areas impair the functional connectivity patterns selectively in the range of alpha.

Relative to the role of alpha oscillatory activity in functional neural connectivity, long-range alpha synchronous activity has been shown to be relevant to promote communication between regions according to task demands (Doesburg et al. 2009; Palva and Palva 2007; Cooke et al. 2019) and coherent alpha oscillations seem to represent one of the main mechanisms of global resting-state integration in the human brain (Guggisberg et al. 2015). Accordingly, alpha cortico-cortical interactions have been suggested to reflect top-down processing, subserving the ability to integrate local bottom-up information (von Stein and Sarnthein 2000). Notably, post-lesional alterations in the alpha band were observed only in intrahemispheric connectivity in hemianopic patients, while alpha interhemispheric connectivity was similar between patients and healthy controls. In line with this finding, where functional connectivity is measured by wPLI, which is an index of lagged phase synchronization, previous evidence has suggested that post-stroke behavioral outcome can be predicted by phase-lagged alpha oscillations in intrahemispheric networks (Guggisberg et al. 2015). In contrast, interhemispheric interactions have shown to be predictive of post-lesional behavior, only when considering amplitude similarities with no phase lag in the beta (Guggisberg et al. 2015).

The present results also highlight the topological features of these post-lesional connectivity changes, showing that the reduced connectivity in upper alpha, is characterized by a decrease in local functional segregation (i.e., clustering coefficient), with no associated change in global functional integration (i.e., characteristic path length). Effective functional segregation has been reported to represent the ability of specific brain regions to integrate all the available information into complex specialized processes, through densely interconnected modules or clusters (Friston 2011). Moreover, network efficiency has typically been described as a balance between this dense local clustering and short path length to integrate information from distant nodes (Sporns 2011; Bassett and Bullmore 2006). Therefore, the observed post-lesional changes in hemianopics suggest a disruption of the optimal brain functional structure, mainly due to a weakened local functional specialization. Previous reports on stroke patients at the acute stage have demonstrated post-lesional decrease in local alpha connectivity processing, which was compensated by a concurrent increase in distant functional integration, suggesting a rearrangement of alpha functional networks, due to plastic compensatory mechanisms occurring immediately after lesions (Caliandro et al. 2017). Following this reasoning, the present findings on hemianopic patients with posterior lesions at the chronic stage, showing an impairment in local alpha functional networks not associated with a concurrent a strengthening of global integration, might represent a signature of the topological dysfunction at the chronic stage after posterior lesions.

In keeping, our results also show a strong association between performance in clinical visual tests and connectivity patterns in the alpha range. Indeed, visual detection performance in the blind field, when eye movements were restricted (Grasso et al. 2016; Passamonti et al. 2009; Bolognini et al. 2005) was positively correlated with connectivity index and clustering coefficient in the upper alpha range. In contrast, no association between alpha connectivity and visual performance, when compensatory eye movements were allowed, was found. These findings suggest a strong link between alpha connectivity patterns after posterior lesions and the basic visual functioning of the visual system (i.e., the size of spared visual field), while more strategic, compensatory visual mechanisms seem not related to these connectivity measures. Accordingly, impaired pattern of alpha functional connectivity has also been observed in patients with visual loss due to pre-chiasmatic lesions (Bola et al. 2014) or retinal damage (Bola et al. 2015). These findings suggest that alterations in alpha functional connectivity during rest can be detrimental for visual performance and, therefore, might represent an index of impairment of the structural and functional integrity of the visual system, reflecting the presence of lesions in visual brain regions or peripheral damage.

Interestingly, right posterior lesions led to a more profound impairment in oscillatory functional connectivity. Indeed, while after left posterior lesions, reduction in intrahemispheric alpha connectivity was found only in the hemisphere ipsilateral to the lesion, in case of lesions of the right posterior cortices the reduction in alpha intrahemispheric connectivity was bilaterally distributed all over the scalp. Moreover, the impairment in alpha connectivity after right posterior lesions was also associated to an abnormal increase in theta connectivity, both intra- and inter-hemispheres. These observations are reminiscent of previous findings revealing at rest that hemianopics with right lesions showed a stronger alpha peak reduction and a more pronounced alpha power interhemispheric imbalance, compared to left-lesioned hemianopics (Pietrelli et al. 2019). Similarly, posterior right lesions also induced a more severe and distributed decrease in alpha reactivity at the opening of the eyes at rest, combined to a disruption in theta reactivity (Gallina et al. 2021).

The presence of more severe and distributed alterations in connectivity patterns in the right-lesioned hemianopic patients may raise the question of whether distinct post-lesional patterns of functional connectivity in the left-lesioned and in the right-lesioned hemianopics also reflect on different outcomes in visual performance. However, to disclose possible hemispheric differences in the patients’ performance, a large sample of patients and specific and sensitive visual tasks, which are not available in the current investigation, are required. Therefore, future studies are needed to further explore differences in post-lesional visual performance depending on the side of the lesion.

Crucially, the more detrimental effects in oscillatory patterns after right posterior lesions seems to suggest a specialization of the right hemisphere in the generation and distribution of alpha oscillations, in line with previous studies consistently reporting a more pronounced alpha activity over the right posterior scalp sites at rest (Metzen et al. 2022). Accordingly, a dominance of the right hemisphere in modulating alpha oscillations, in order to allocate visuo-spatial attentional resources and tune visual perceptual abilities, has also been demonstrated during spatial orienting tasks with directional cues in healthy individuals (Gallotto et al. 2020).

Notably, although previous reports have documented post-lesional increased connectivity in low frequencies (delta-theta; Dubovik et al. 2012; Castellanos et al. 2010), the present findings seem to suggest that altered theta connectivity might be linked to specific lesional profiles, involving posterior cortices in the right hemisphere, and is consistently associated to impairments also in alpha connectivity. Moreover, graph theoretical analyses revealed that theta connectivity impairment is characterized by an increase in local segregated activity (i.e. increased clustering coefficient) and a decrease in global integration (i.e., higher path length), suggesting both a local and global inefficiency in theta activity. This suggests that a severe and bilateral reduction in alpha connectivity (as observed in right-damaged hemianopics) also induces impairments in local and global low-level processing in the theta range. In line, alpha oscillatory activity is thought to reflect widespread cortical networks’ activity, regulating modular processes (Barry and De Blasio 2017; Doesburg et al. 2009) and orchestrating oscillatory activity in different frequency bands (Hindriks et al. 2015). In this perspective, we can speculate that in case of left posterior lesions, spared activity in the right intact hemisphere, which shows normal alpha connectivity patterns, might be sufficient to preserve normal connectivity also in the theta range. On the contrary, right posterior lesions disrupt such oscillatory regulatory mechanisms, leading to functional connectivity impairments also in the theta range, therefore, suggesting that the right hemisphere might have a pivotal role in this mechanism. This seems in agreement with a longstanding range of evidence reporting a dominance of the right hemisphere in perceptual and visuo-spatial processing (Bueichekú et al. 2020; Corballis et al. 2002; Nicholls and Roberts 2002; Jewell and McCourt 2000; Gitelman et al. 1999; McCourt and Jewell 1999; McCourt and Olafson 1997; Nobre et al. 1997; Mattingley et al. 1994; Heilman et al. 1984; Heilman and Valenstein 1979; Bisiach and Luzzatti 1978) and corroborate the notion that oscillatory mechanisms might play a role in this specialized function.

Overall, the present results suggest that different lesional profiles might differentially alter functional connectivity and show that lesions to posterior cortices specifically impair functional connectivity in the alpha range. This suggests that the connectivity in the alpha range might represent an index of the integrity of the underlying visual system and supports the role of alpha oscillations in regulating local and global oscillatory patterns in lower frequency bands.

To conclude, there is accumulating evidence converging on the notion that the alpha oscillatory patterns at rest may represent a reliable signature of the functional and structural dynamics of the damaged visual system. Importantly, alpha oscillatory activity is known to be susceptible to functional changes and can be effectively modulated with external stimulations through various non-invasive brain techniques, such as transcranial magnetic stimulation (TMS), transcranial alternating current stimulation (tACS), rhythmic sensory entrainment methods or neurofeedback. This holds promise for future studies to develop effective therapeutic interventions for patients with posterior brain lesions, targeting alpha oscillatory power, frequency and functional connectivity at rest, to promote the functioning of the visual system and to ameliorate visual abilities in hemianopic patients (Dundon et al. 2015a, b).

## Data Availability

The datasets generated during and/or analyzed during the current study are available from the corresponding author on reasonable request.

## Code availability

Custom codes used for analyses are available from the corresponding author on reasonable request.
